# Metabolic Visualization Reveals the Distinct Distribution of Sugars and Amino Acids in Rice *Koji*

**DOI:** 10.5702/massspectrometry.A0089

**Published:** 2020-08-26

**Authors:** Adinda Putri Wisman, Yoshihiro Tamada, Shuji Hirohata, Eiichiro Fukusaki, Shuichi Shimma

**Affiliations:** 1 Department of Biotechnology, Graduate School of Engineering, Osaka University; 2 HAKUTSURU SAKE Brewing Co., Ltd.; 3 Osaka University Shimadzu Analytical Innovation Laboratory, Osaka University

**Keywords:** rice *koji*, mass spectrometry imaging, sugars, amino acids

## Abstract

The compounds inside rice *koji* have been thoroughly investigated as an essential material in making many food-related products, including *sake*. However, these studies focused only on quantitative aspects, leaving features that can still be uncovered if seen from a new perspective. Visualization of the metabolites inside rice *koji* may as well be the new angle needed to retrieve more information regarding rice *koji* making. Here we utilized mass spectrometry imaging (MSI) to visualize the distribution of sugars, sugar alcohols, and amino acids inside rice *koji*. Imaging results revealed that several sugars alcohols and amino acids were shown to have characteristic distribution near the edges or surface of rice *koji*. Furthermore, the distribution appears to be correlated with the different structure of rice *koji*. This study is the first report of using MSI to visualize sugars, sugar alcohols, and amino acids in rice *koji*.

## INTRODUCTION

In the world of *sake* brewing, rice *koji* is considered as an essential material for being an enzyme source in the main fermentation with yeast. Rice *koji* is made by inoculating steamed rice with the *koji* mold, *Aspergillus oryzae* (*A. oryzae*). The mold propagation on steamed rice promotes the production of amylolytic and proteolytic enzymes as well as various metabolites.^1)^ As such, the quality of *koji* is generally determined by its enzyme activity and its components that affect the quality of the *sake*.^2)^

Many types of research have investigated the content of *koji*, as well as factors that affect the change in *koji* quality, such as fermentation time,^3)^ rice polishing rate,^4)^ as well as the strain of fungi used.^5,6)^ All of the studies rely on the quantitative aspect and increase of sugars, sugar alcohols, and amino acids were generally observed. While the information obtained has been proved useful for improvement in production, many aspects are still not well understood. As such, another perspective is required to help gain insight into the process of rice *koji* making.

Mass spectrometry imaging (MSI) is a rising analytical method for the analysis of foodstuffs.^7,8)^ This technique can visualize multiple compounds directly without labeling, unlike other imaging techniques. It has been used to visualize many components in food, such as lipids,^9)^ sugars, organic acids,^10)^ and secondary metabolites.^11,12)^ The application of MSI on various food products has opened up new findings and add new insight on various aspects, such as food safety and characterization.

Our previous study has demonstrated the possible correlation between glucose localization and mycelial penetration.^13)^ However, the distribution of other components related to amylolytic and proteolytic enzymes inside rice *koji* may give more information on the changes that happen during the rice *koji* making process. With mycelial penetration involved in the nature of rice *koji*-making, there is a possibility that certain metabolites will exhibit different distribution profiles even when they show the same increasing trend quantitatively. In this study, we performed the MSI analysis on steamed rice and rice *koji* to obtain the distribution profile of sugars, sugar alcohols, and amino acids inside rice *koji* for the first time.

## EXPERIMENTAL

### Samples and reagents

*Yamadanishiki*-type (polishing rate 70%) steamed rice and rice *koji* samples were acquired from the mainline production in Hakutsuru Sake Brewing Co., Ltd. The rice *koji* were collected 42 h after fermentation. Standards (sugars, sugar alcohols and amino acids) were purchased from FUJIFILM Wako Pure Chemical Corporation (Osaka, Japan), Merck & Co., Inc. (Rockville, MD, USA), Nacalai Tesque, Inc. (Kyoto, Japan), or Tokyo Chemical Industry Co., Ltd. (Tokyo, Japan). For the preparation of solvents, methanol (MeOH) was purchased from Kanto Chemical Co., Inc. (Tokyo, Japan), while ultrapure water was obtained through Barnstead GenPure xCAD Plus water purification system by Thermo Fisher Scientific (Tokyo, Japan). The matrix *N*-(1-naphthyl)ethylenediamine dihydrochloride (NEDC), 2,5-dihydroxybenzoic acid (DHB), and derivatization agent for amino acid imaging 2,4-diphenyl-pyranylium tetrafluoroborate (DPP-TFB) were purchased from Merck. Triethylamine (TEA) for part of the DPP-TFB buffer was obtained from Wako.

### Sample preparation for mass spectrometry imaging

Sample sectioning was conducted based on the previous report.^13)^ Serial sections of each sample were obtained and attached to Indium Tin Oxide (ITO) glass (100 Ω/m^2^ without anti-peeling coating; Matsunami Glass, Osaka, Japan) using conductive double-sided tape (Shielding Non-woven Fabric Tape; 3 M company St. Paul, MN, USA). For sugar imaging, a solution of the NEDC matrix (7 mg/mL in 50% MeOH) was applied manually using an airbrush (PS-270, GSI Creos, Tokyo, Japan). For amino acid imaging, the DPP-TFB stock solution (10 mg/mL in MeOH) was first prepared. The working solution for derivatization contained 8% of the stock solution in a buffer containing MeOH, ultrapure water, and TEA (6 : 9 : 0.01, v/v/v). The samples were sprayed with the DPP-TFB working solution and incubated for 1.5 h. The matrix DHB (50 mg/mL in 50% MeOH) was then subsequently sprayed before analysis.

### Mass spectrometry imaging analysis

Mass spectrometry imaging was performed using iMScope TRIO (Shimadzu, Kyoto, Japan). For sugar analysis, the instrument was operated in negative mode, and spectra were acquired in the range of *m*/*z* 115 to 550. One section of each sample was used for the scan analysis. For amino acid imaging, the instrument was operated in positive mode, and the fragment ion *m*/*z* 232.11 were targeted for each amino acid precursor. Two serial sections of each sample were used for the targeted MS/MS analysis. Each section was used to analyze nine targeted amino acids. The area of laser shot was shifted 10 μm to either right or downwards of the original area after each analysis. Laser diameter and intensity was set to 2 (arbitrary unit; approximately 25 μm) and 70 (arbitrary unit), respectively. All MSI images were acquired with a 50 μm pitch. Acquired data were then processed using Imaging MS Solution (Shimadzu, Kyoto, Japan). For the comparison of distribution, absolute intensity from the sample with higher value of each metabolites were used as the high limit value for both samples.

## RESULTS AND DISCUSSION

### Visualization of sugars by mass spectrometry imaging

Rice *koji*-making induces the production of amylolytic enzymes, resulting in the conversion of starch into simple sugars. Monosaccharides such as glucose, fructose, galactose as well as disaccharides such as sucrose and maltose are commonly found in rice *koji*.^4,5)^ The increase of sugar alcohols such as erythritol, arabitol, and xylitol after rice *koji*-making were also previously reported.^5,6)^ In this study, different types of sugars, such as monosaccharide and disaccharide, as well as sugar alcohols, were targeted for MSI analysis. However, isomers were not able to be separated using this method, causing some sugars that share the same *m*/*z* value could not be distinguished.

In sugar imaging, the NEDC matrix was utilized as it was reported to be able to visualize glucose due to the formation of [M+Cl]^−^ adducted ion.^14)^ This principle was known to apply to other sugars.^15)^ Hence, other sugars are theoretically able to be detected with the same method. Detection was confirmed by analyzing sugar and sugar alcohol standards mixed with the NEDC matrix (Supplementary Fig. S1).

**Figure 1:**
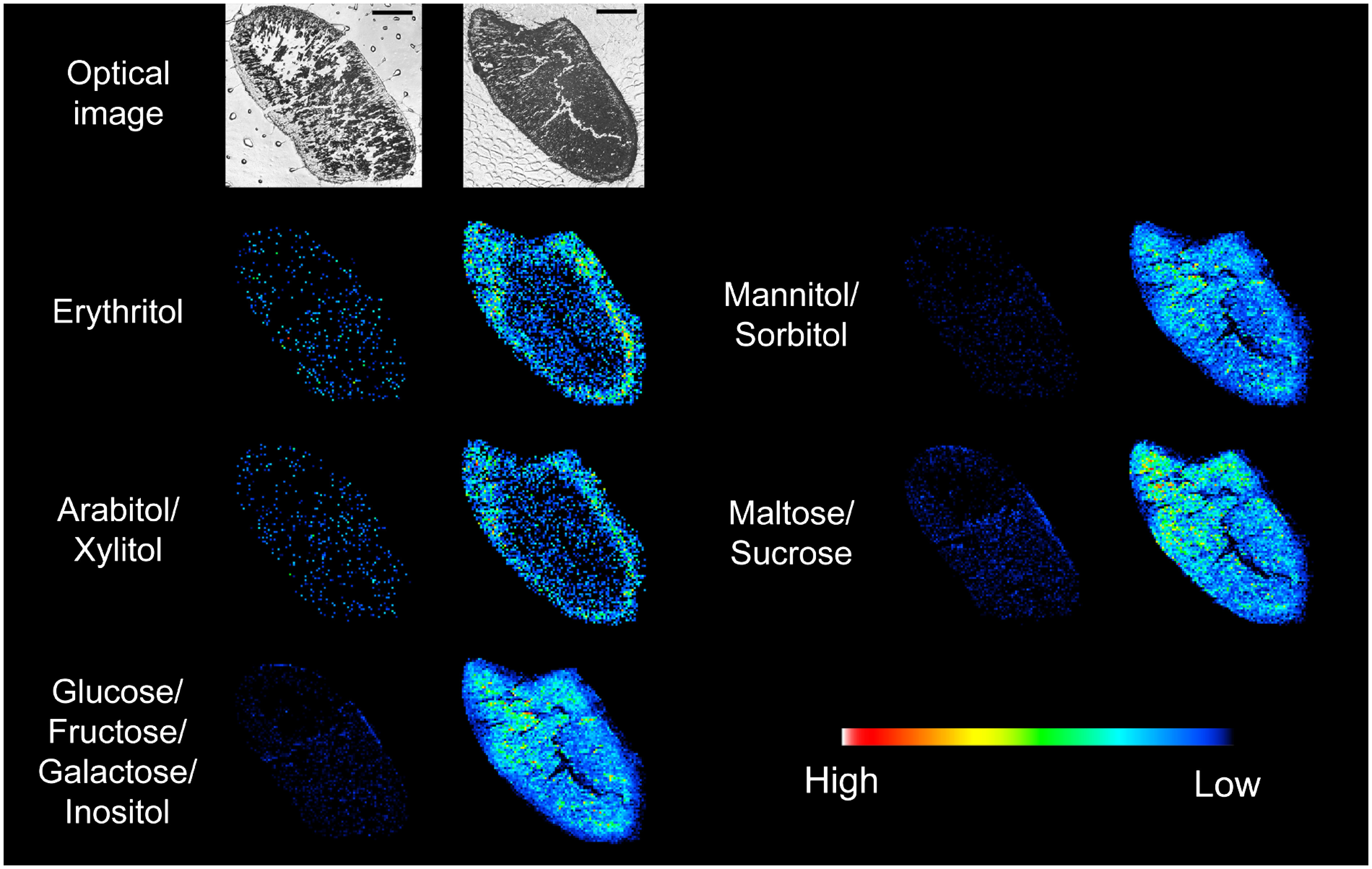
Fig. 1. Ion intensity distribution of detected sugars and sugar alcohols in steamed rice and rice *koji*. The metabolites are erythritol (*m*/*z* 157.03), arabitol/xylitol (*m*/*z* 187.04), glucose/fructose/galactose/inositol (*m*/*z* 215.03), mannitol/sorbitol (*m*/*z* 217.04), and maltose/sucrose (*m*/*z* 377.05). Optical images of steamed rice and rice *koji* sections accompanies the imaging results. Scale bar is 1 mm.

The method was then applied to imaging by spraying the NEDC matrix on the sample. Sugar and sugar alcohols were all detected as [M+Cl]^−^ ion (Fig. 1). There are 5 peaks detected; *m*/*z* 157 (erythritol), *m*/*z* 187 (arabitol, xylitol), *m*/*z* 215 (fructose, galactose, glucose, inositol), *m*/*z* 217 (mannitol, sorbitol), and *m*/*z* 377 (maltose, sucrose). The peak detected at *m*/*z* 215 are thought to be mainly originating from glucose, as the major sugar produced by the amylolytic enzymes originating from rice *koji* is reported to be glucose.^16)^ However, the possibility of glucose isomers also included in the distribution profile of *m*/*z* 215 should be acknowledged.

Based on the relative intensity, all sugars and sugar alcohols were detected higher in rice *koji*. Most of the peaks showed even distribution on rice *koji*, apart from *m*/*z* 157 and *m*/*z* 187, which shows accumulation near the edge of rice *koji*. These peaks are derived from the sugar alcohols erythritol as well as arabitol and/or xylitol. These sugar alcohols may be derived from hemicellulose abundant in rice grains, especially in the bran layer. However, the rice used in this study has 70% polishing rate, and is believed to have been removed completely of the bran layer.^17)^ On the other hand, it has been reported that filamentous fungi such as *A. oryzae* tend to accumulate polyols or sugar alcohols at a low water activity (*a*_w_) environment.^18)^ Rice *koji* making is a type of solid-state fermentation (SSF), and as such, has a low *a*_w_ environment. The fungi prevent water loss by accumulating compatible solutes such as polyols. Previous studies have shown the tendency of *Yamadanishiki* rice to accumulate water in the core of rice grain instead of the surface.^19,20)^ The high accumulation of these polyols on the surface of rice *koji* may be due to low *a*_w_ in that area compared to the inside of rice. Another theory that could be generated is that the fungus produced polyols to function as a “barrier” to prevent the moisture inside from drying out. In this case, the profile of mycelial growth in rice *koji* may provide additional perspective in future studies.

On the other hand, not all sugar alcohols seem to accumulate on the edges, such as inositol, mannitol, and sorbitol. Inositol distribution could not be differentiated due to being an isomer with other monosaccharides such as glucose, which has *m*/*z* 215. In addition, mannitol and sorbitol at *m*/*z* 217 appeared to have the same distribution pattern as glucose at *m*/*z* 215. There is a possibility that the *m*/*z* 217 peak is mainly detected as an isotope of *m*/*z* 215, as this peak also appeared on standard detection of glucose and other sugars sharing the same *m*/*z* value.

### Visualization of amino acids by mass spectrometry imaging

During the rice *koji*-making process, the *koji* mold produces proteolytic enzymes to obtain nitrogen necessary for growth. The change of amino acid content after *koji* fermentation has been reported by several studies.^4,21)^ In this study, the distribution of amino acids inside rice *koji* was investigated (Supplementary Table S2). However, several amino acids with isomers such as leucine and isoleucine were not able to be separated using this method.

**Figure 2:**
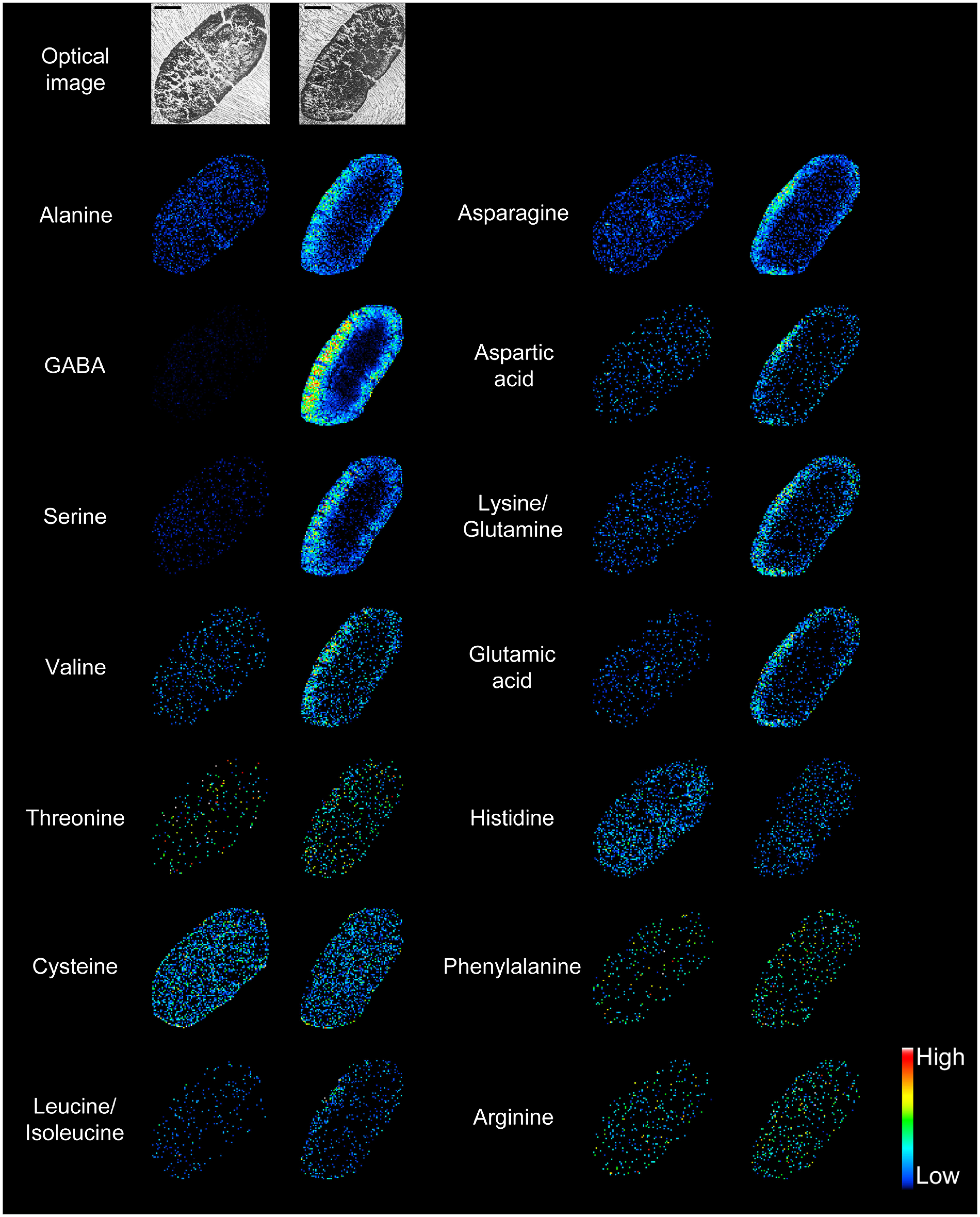
Fig. 2. Ion intensity distribution of detected amino acids in steamed rice and rice *koji*. The amino acids are alanine (*m*/*z* 304.18), GABA (*m*/*z* 318.21), serine (*m*/*z* 320.18), valine (*m*/*z* 332.24), threonine (*m*/*z* 334.21), cysteine (*m*/*z* 336.15), leucine/isoleucine (*m*/*z* 346.26), asparagine (*m*/*z* 347.21), aspartic acid (*m*/*z* 348.20), lysine/glutamine (*m*/*z* 361.20), glutamic acid (*m*/*z* 362.22), histidine (*m*/*z* 370.17), phenylalanine (*m*/*z* 380.28), and arginine (*m*/*z* 389.21). Ion intensity distribution shown are from the fragment peak *m*/*z* 232.11 of each precursor ion peaks. Optical images of steamed rice and rice *koji* sections accompanies the imaging results. Scale bar is 1 mm.

In amino acid imaging, derivatization agent DPP-TFB was applied before matrix application. On-tissue detection of amino metabolites has been known to be difficult due to the low-ionization efficiency and spectral interferences from the MALDI matrix. Thus, the use of on-tissue derivatization is one of the methods to increase amino metabolites detectability.^22)^ Derivatized amino acids were detected through MS/MS analysis as *m*/*z* 232.11 after fragmentation of the parent ions (Fig. 2). There are 14 peaks detected; *m*/*z* 304 (alanine), *m*/*z* 318 (GABA), *m*/*z* 320 (serine), *m*/*z* 332 (valine), *m*/*z* 334 (threonine), *m*/*z* 336 (cysteine), *m*/*z* 346 (leucine, isoleucine), *m*/*z* 347 (asparagine), *m*/*z* 348 (aspartic acid), *m*/*z* 361 (lysine, glutamine), *m*/*z* 362 (glutamic acid), *m*/*z* 370 (histidine), *m*/*z* 380 (phenylalanine), and *m*/*z* 389 (arginine). Other amino acids, such as glycine, proline, tyrosine, and tryptophan, were also investigated. While glycine was able to be detected, the signals were accumulated on blank spots of the samples (data not shown), leading us to believe that it was not the real distribution of glycine. The remaining amino acids, on the other hand, had a very low signal intensity and thus were hard to visualize.

In general, all amino acids were shown to have higher intensity in rice *koji* relative to steamed rice except for cysteine (*m*/*z* 336) and histidine (*m*/*z* 370). Furthermore, the distribution profile on rice *koji* differed into two patterns. The first pattern exhibited accumulation around the edges (alanine, GABA, serine, valine, leucine/isoleucine, asparagine, aspartic acid, lysine/glutamine, glutamic acid), while the other amino acids (threonine, cysteine, histidine, phenylalanine, arginine) were distributed evenly on the cross-section of rice *koji*. The accumulation of amino acids near the surface of rice *koji* could be related to how most proteins in rice are near the surface. A previous study mentioned that rice with a low polishing rate contains more protein, and a higher polishing rate will result in lower protein content.^23)^ This implies that the proteins are mostly accumulated away from the center of the rice. The rice used in this study had a low 70% polishing rate, which means most of the proteins would be concentrated on the edges of rice. Another study using 70% polishing rate *Yamadanishiki* rice also showed how the storage proteins are not accumulated in the center of the grain,^24)^ backing up this hypothesis.

The remaining amino acids (threonine, cysteine, histidine, phenylalanine, and arginine) were shown to have an even distribution on the cross-section of rice *koji*. Cysteine was decreased after *koji* fermentation, implying that *A. oryzae* mainly utilized the cysteine that was originally already in rice. A previous study also mentioned that the production of cysteine during rice *koji* making is lower than other amino acids.^21)^ The different distribution with other amino acids may suggest that these amino acids have a different role to *A. oryzae* growth in SSF. However, current results were not able to provide enough information to explain this phenomenon.

On the other hand, the apparent difference in intensity trend between the previous study and visualization of histidine was observed. It has been reported that rice already contained histidine,^25)^ while the production of histidine by *A. oryzae* was not as high as other amino acids.^21)^ There is a possibility that the steamed rice sample used in this study already had a high amount of histidine. In contrast, the rice *koji* sample used has originally low histidine from the rice as well as low production from the fungi itself.

**Figure 3:**
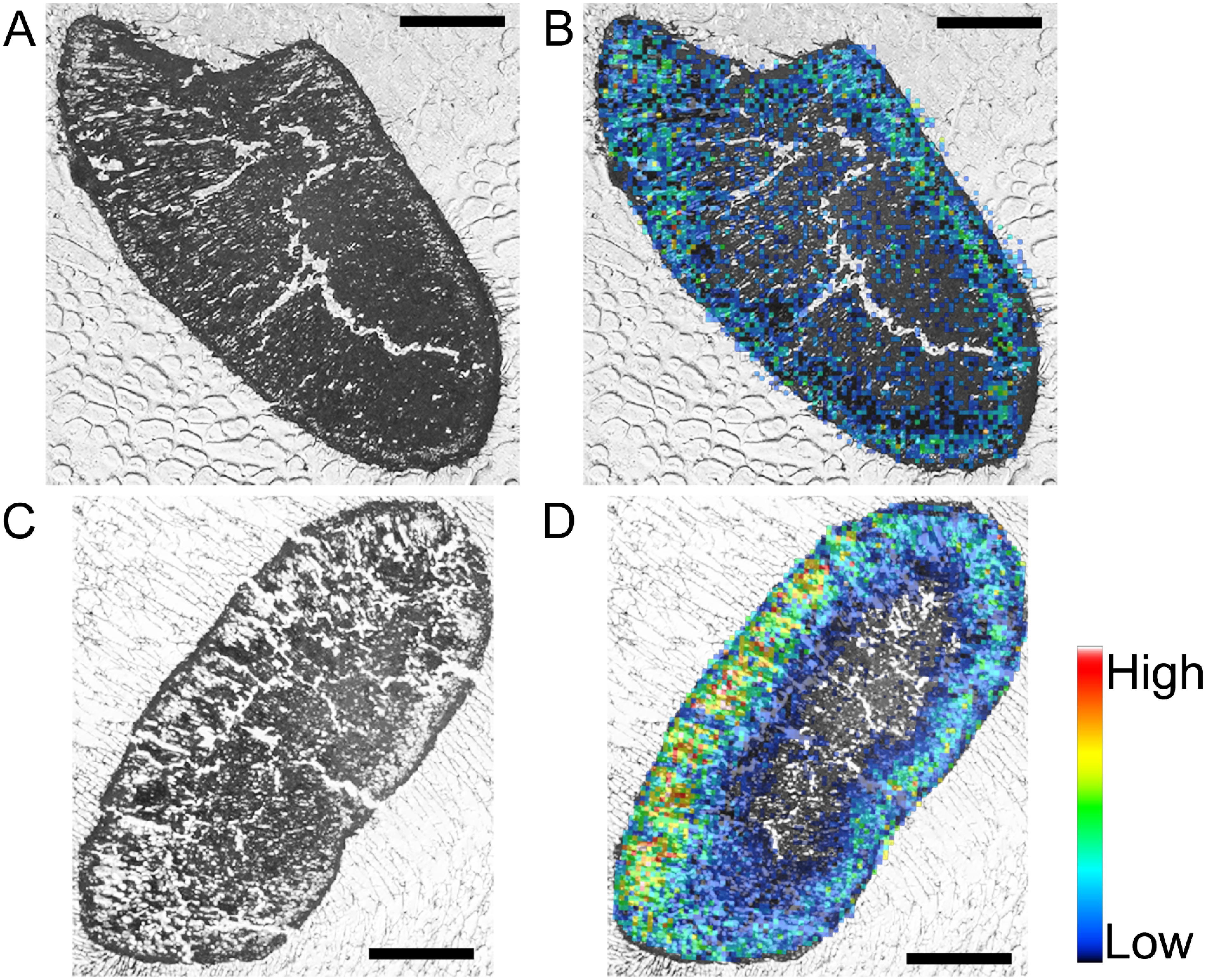
Fig. 3. Observation of rice *koji* cross-section (A, B) corresponding to the distribution of sugar alcohol representative, arabitol/xylitol (*m*/*z* 187.04) (C), and amino acid representative, GABA (*m*/*z* 318.21, visualized by fragment peak *m*/*z* 232.11) (D). Scale bar is 1 mm.

At a more detailed observation, the edges of rice *koji* observable from the optical image showed some white parts that were not related to the cracks in the section. These parts corresponded with the accumulation of amino acids as well as several sugar alcohols on the edges of rice *koji* (Fig. 3). The difference in color may signify a different form of steamed rice cell that may be changed due to *A. oryzae* growth. It was also reported that a collapsed structure inside rice *koji* indicates that the fungi have penetrated and digested the rice for nutrition.^26)^ The white-colored parts may as well be the collapsed structure mentioned. However, visualization of mycelial growth may be needed to prove this theory. Nevertheless, the fact that several metabolites distribution were corresponding with the change in rice structure after rice *koji* making raises the possibility of *A. oryzae* growth profile being important to metabolites accumulation.

The distribution profile of sugars, sugar alcohols, and amino acids in rice *koji* was investigated through mass spectrometry imaging analysis. Previous studies have explored the change in the metabolic profile during rice *koji* making. This study confirmed the findings as well as adding another perspective by visualizing several changed metabolites distribution in rice *koji*. MSI analysis revealed unique distribution depending on the metabolites observed, a new finding that could not be obtained using conventional methods. Furthermore, the distribution profile seemed to be related to the different structure of rice *koji*. The new information provided by the imaging results opened possibilities in understanding not only the process happening during rice *koji* making process, but also the growth characteristic of *Aspergillus* fungi in general SSF processes.

## Notes

Mass Spectrom (Tokyo) 2020; 9(1): A0089
