# Supplementary material for: Metabolic Visualization Reveals the Distinct Distribution of Sugars and Amino Acids in Rice Koji
Source: Mass Spectrom (Tokyo). 2020 Aug 26;9(1):A0089. doi: 10.5702/massspectrometry.A0089 (PMC7471875; doi:10.5702/massspectrometry.A0089)
Supplement: Supplementary Data [file massspectrometry-9-1-A0089-s001.pdf]

Supplementary Data

Metabolic visualization reveals the distinct distribution of sugars and amino acids in rice *koji*

Adinda Putri Wisman,[1] Yoshihiro Tamada,[2] Shuji Hirohata,[2] Eiichiro Fukusaki,[1,3] Shuichi Shimma[1,3]*

[1]*Department of Biotechnology, Graduate School of Engineering, Osaka University, 2-1 Yamadaoka, Suita, Osaka, 5650871, Japan*

[2]*HAKUTSURU SAKE Brewing Co., Ltd., 4-5-5 Sumiyoshi Minamimachi, Higashinada-ku, Kobe, Hyogo, 6580041, Japan*

[3]*Osaka University Shimadzu Analytical Innovation Laboratory, Osaka University, 2-1 Yamadaoka, Suita, Osaka, 5650871, Japan*

***Corresponding author:** S. Shimma

(Tel and Fax) +81-6-6879-7418, (E-mail address) sshimma@bio.eng.osaka-u.ac.jp

Table S1. List of detected sugars and sugar alcohols from MSI analysis

No	Sugars	Theoretical MW	Theoretical *m/z* after Cl ion adduct	Detected *m/z* value
1	Erythritol	122.12	156.98	157.03
2	Arabitol	152.14	187.00	187.04
3	Xylitol	152.15	187.01	
4	Fructose	180.16	215.02	215.03
5	Galactose	180.16	215.02	
6	Glucose	180.16	215.02	
7	Inositol	180.16	215.02	
8	Mannitol	182.17	217.03	217.04
9	Sorbitol	182.17	217.03	
10	Maltose	342.30	377.16	377.05
11	Sucrose	342.30	377.16	

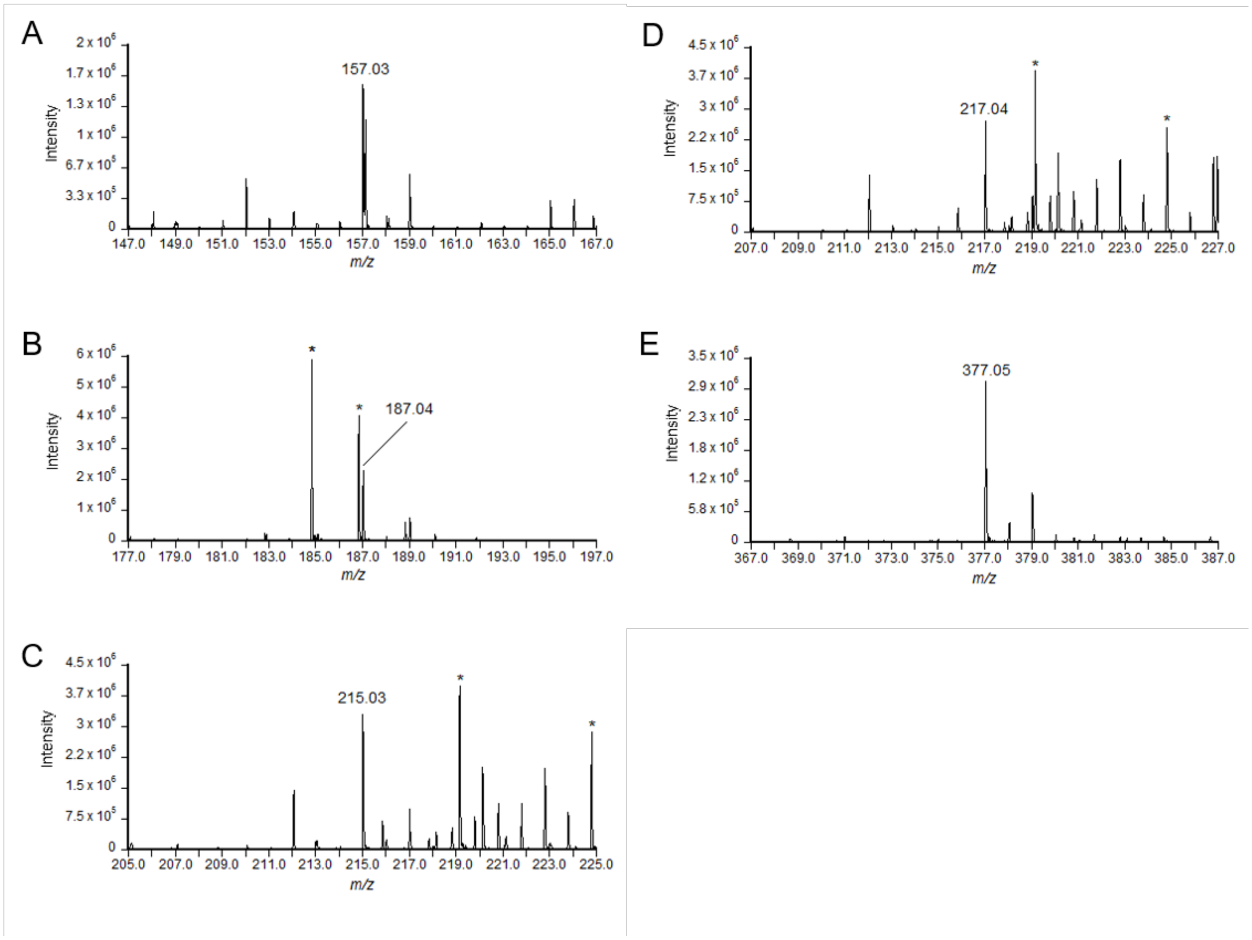

Fig. S1. Mass spectra of sugars and sugar alcohols acquired from standards. (A) *m/z* 157 (represented by erythritol), (B) *m/z* 187 (represented by arabitol), (C) *m/z* 215 (represented by glucose), (D) *m/z* 217 (represented by mannitol), and (E) *m/z* 377 (represented by sucrose). Asterisks represent peaks derived from the NEDC matrix. Intensity is in arbitrary unit.

Table S2. List of detected amino acids from MSI analysis

No	Amino acids	Theoretical MW	Theoretical *m/z* after DPP-TFB derivatization
1	Alanine	89.09	304.18
2	GABA	103.12	318.21
3	Serine	105.09	320.18
4	Valine	117.15	332.24
5	Threonine	119.12	334.21
6	Cysteine	121.06	336.15
7	Isoleucine	131.17	346.26
8	Leucine	131.17	
9	Asparagine	132.12	347.21
10	Aspartic acid	133.11	348.20
11	Lysine	146.11	361.20
12	Glutamine	146.14	
13	Glutamic acid	147.13	362.22
14	Histidine	155.08	370.17
15	Phenylalanine	165.19	380.28
16	Arginine	174.12	389.21
